# Semi-Analytical Investigation into the Balanced Performance of Thick-Walled Fiber-Reinforced Flexible Pipes

**DOI:** 10.3390/ma19051007

**Published:** 2026-03-05

**Authors:** Jingyue You, Yinglong Zhao, Ben Zhang

**Affiliations:** Naval University of Engineering, Wuhan 430030, China

**Keywords:** thick-walled, fiber-reinforced flexible pipe, balanced performance, principle of minimum potential energy, Newton-Raphson method

## Abstract

The balanced performance of fiber-reinforced flexible (FRF) pipes is essential for maintaining dimensional stability and structural integrity in pipelines. However, current theoretical approaches face challenges in simultaneously incorporating end effects, geometric nonlinearity, and material nonlinearity, resulting in a persistent reliance on engineering experience when determining balanced fiber winding angles. This work proposes a semi-analytical method for evaluating the balanced performance of thick-walled FRF pipes, based on the strain energy density function, with governing equations established by integrating finite deformation theory and the principle of minimum potential energy. A displacement trial function is adopted to approximate the actual displacement field, with its coefficients determined iteratively using the Newton–Raphson method. An eight-coefficient displacement trial function demonstrates effectiveness in characterizing the pipe’s deformation characteristics under the maximum working internal pressure, capturing key deformation features such as radial inward expansion with outward restraint gradient, nonlinear axial deformation, and axial end warping. The proposed method is validated against both experimental results and finite element simulations, and an analysis of the fiber winding angle’s influence on balanced performance is conducted, thereby establishing a theoretical basis for the design of self-balanced thick-walled FRF pipes.

## 1. Introduction

Fiber-Reinforced Flexible (FRF) pipes are composite structures composed of polymer matrices and reinforcing fibers. Polymer matrices function not only as an adhesive that immobilizes reinforcing fibers but also contribute critically to load transfer, resistance to physical damage, and protection against chemical corrosion. Reinforcing fibers, serving as the primary load-bearing elements, significantly enhance the pipe’s overall mechanical performance due to their high specific strength and high modulus [1,2,3]. The flexibility in selecting both matrix and fiber materials, along with structural design adaptability, enables tailored performance of FRF pipes, allowing them to meet diverse application requirements in fields such as marine and civil engineering [4,5,6].

FRF pipes can be installed as expansion joints at connection points between equipment and pipelines or between different pipeline segments [7]. Compared with traditional metal pipes in pipeline systems, FRF pipes, with their low stiffness and high flexibility, not only function as elastic buffer elements [8], but also allow effective displacement compensation under large deformations and complex loading conditions [9]. Furthermore, by optimizing the fiber winding angle, FRF pipes can achieve balanced performance. Self-balanced FRF pipes possess the capability to autonomously regulate axial deformation induced by internal pressure, enabling the pipes to actively counteract axial strain during pressurized media transport. This characteristic prevents the generation of additional thrust forces or constrained displacements on connected equipment and pipelines [10].

As established in the theory of thin-walled cylinders, the ratio of hoop stress to axial stress is 2:1 for a cylinder with closed ends. According to grid analysis theory [11,12], if reinforcing fibers in an FRF pipe carry tensile loads exclusively along their axial direction, material efficiency can be maximized and deformation in the non-fiber directions can be effectively suppressed. Consequently, when the fiber winding angle is set to $\arctan\left(\sqrt{2}\right)$, the fiber orientation aligns with the principal stress direction of the pipe. Under this condition, the axial strain induced by internal pressure can be counteracted, enabling the thin-walled pipe to achieve a self-balanced state. Therefore, in Gao et al.’s study [13] on the structural design of self-balanced FRF pipes, their theoretical model accounted only for the mechanical contribution of reinforcing fibers. However, this theory relies on the assumption that reinforcing fibers dominate the structural behavior. In reality, the mechanical performance of the reinforcement layer arises from the combined contributions of both reinforcing fibers and polymer matrices within the layer.

The mechanical properties of the reinforcement layer essentially depend on the distribution, orientation, geometric morphology and volume fraction of the reinforcing fibers and matrix, as well as the interface characteristics and bonding state formed during the manufacturing process [14]. To characterize the mechanical properties of such composites, homogenization techniques are commonly adopted [15]. For short-fiber-reinforced composites, Chao et al. [16] proposed an asymptotic homogenization strategy based on local–global representative volume elements, which can effectively predict the elastic properties of random fiber-reinforced composites with high fiber volume fractions. In multi-scale homogenization research, the interface debonding effect between fibers and the matrix has been gradually considered. For instance, Heide-Jorgensen et al. [17] established a three-dimensional multi-scale homogenization method for hybrid woven composites, which can be used to calculate the orthotropic material parameters considering interface damage. In addition, for fiber-reinforced composites under large deformation conditions, Li et al. [18] constructed a large-deformation mean-field homogenization model, which can be embedded into ABAQUS through a user material interface and accurately characterize the hyperelastic and elastoplastic homogenized mechanical responses of materials. Within the framework of variational principles and a virtual work statement, Islam et al. [19] proposed an energy-based reduced-order model for hyperelastic materials reinforced with unidirectional and bidirectional fibers, and refined continuum-based prediction models to characterize the nonlinear responses of the matrix and fibers. The governing equations and boundary conditions were derived through variational principles and verified by experiments, which can predict the strain-stiffening behavior of elastomer-polyester fiber composites.

In the theoretical study on the mechanical behavior of filament-wound pipes, the linear equivalent elastic constants of the reinforcement layer are usually first obtained through homogenization, and the stiffness matrix in the principal coordinate system is established, and then transformed into the cylindrical coordinate system for analysis. For example, Rosenow [20] employed micromechanics theory to predict the equivalent mechanical properties of the reinforcement layer and further integrated classical laminated plate theory to analyze the influence of the fiber winding angle on the stress–strain behavior of glass fiber–reinforced polyester filament-wound pipes under internal pressure. Xia et al. [21,22] also adopted micromechanics theory and coupled it with three-dimensional anisotropic elasticity theory based on the generalized Hooke’s law to derive an exact elastic solution for the stress and deformation of multi-layered filament-wound composite pipes under internal pressure. Their results demonstrate that the stress state and deformation behavior of the pipe are highly sensitive to the fiber winding angle. Based on these studies, Zhou et al. [4] developed a systematic programming approach that enables parametric analysis of multi-layered reinforced pipes through iterative loops and conditional control logic, performing failure analysis on a fourteen-layer FRF pipe.

**Figure 1:**
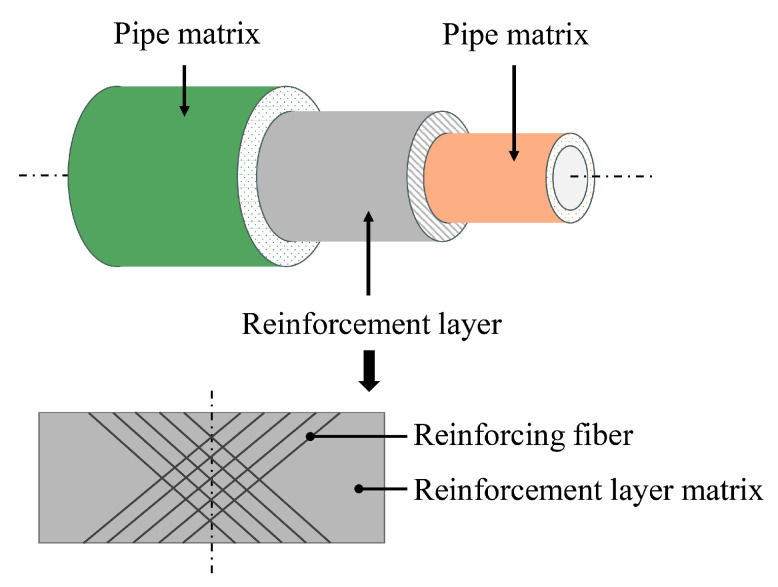
Structural composition of fiber-reinforced flexible (FRF) pipes.

The above studies, while accounting for the composite effect of fibers and matrices within the reinforcement layer, have overlooked the mechanical contribution of the independent pipe matrix (as shown in Figure 1). Reference [12] indicates that the approximation of neglecting the pipe matrix’s contribution is valid only when its stiffness is significantly lower than that of the reinforcing fiber. However, there is currently no established criterion specifying the stiffness ratio between the pipe matrix and the reinforcing fiber across different material combinations, and the threshold range of stiffness ratios beyond which the pipe matrix influence becomes negligible remains undefined. Furthermore, to meet the increasing demand for transporting fluids under higher working pressures, FRF pipes are often designed with higher thickness-to-diameter ratios [23]. This thick-walled configuration results in non-uniform stress distribution and complex interlayer coupling effects, thereby necessitating the inclusion of the pipe matrix in mechanical modeling.

To more comprehensively account for the influence of the pipe matrix, Gu et al. [24] incorporated the rubber material constituting the pipe matrix into the model based on the theories in References [21,22], and investigated the response of an infinitely long FRF pipe under internal pressure. Through experimental validation, they demonstrated that the discrepancy between theoretical predictions and measurements mainly originated from the simplification of rubber as a linear elastic material. Clearly, to accurately describe the mechanical behavior of polymer pipe matrices such as rubber, the material nonlinearity must be taken into account [25]. However, existing theoretical approaches face inherent limitations when modeling nonlinear stress–strain relationships of materials. For example, the method reported in Reference [26] requires iterative updating of the Young’s modulus at each incremental step, which not only increases computational complexity but also restricts the simulation to nonlinear responses under single-mode deformation. Consequently, such methods are generally inadequate for predicting the behavior of hyperelastic materials exhibiting multiaxial stress coupling under complex loading conditions.

Meanwhile, the finite element method has been widely employed to address material nonlinearity issues in the analysis of FRF pipes [27,28,29]. However, numerical methods generally entail high computational costs and are not well suited for directly elucidating the parametric influence mechanisms. Moreover, during the early stages of self-balanced pipes’ design, the finite element method is inconvenient for efficiently identifying the optimal fiber winding angle that enables pipes to achieve the self-balanced state.

Assessing the balanced performance of thick-walled FRF pipes under the maximum working internal pressure requires accurate calculation of the axial deformation. If the axial deformation falls within the engineering allowable range (typically ±1 mm), the pipe is considered to exhibit balanced performance. However, existing theoretical studies generally adopt the assumption of an infinitely long pipe to facilitate closed-form solutions, under which condition the axial strain is treated as constant, thereby precluding the prediction of actual axial deformation. Meanwhile, the thick-walled design commonly implemented in engineering to enhance pressure resistance further amplifies the influence of end constraint effects and the mechanical contribution of the pipe matrix. Furthermore, accurately characterizing the nonlinear stress–strain behavior of the polymer matrix is critical to achieving high prediction accuracy in mechanical models. These limitations hinder the precise description of the mechanical response of thick-walled FRF pipes, resulting in the determination of the balanced fiber winding angle still relying on engineering experience.

In summary, this work formulates the total potential energy using the strain energy density function, employs parameterized displacement trial functions to approximate the actual displacement field, and converts the original boundary value problem into a multivariate extremum problem within the framework of energy principles and reduced-order modeling. By integrating the Newton–Raphson method, the coefficients of the displacement trial functions are iteratively determined. The present approach is established as a semi-analytical reduced-order model based on energy principles and parametric displacement field approximation, aiming to reveal the intrinsic mechanism underlying the balanced performance of thick-walled FRF pipes and to provide a theoretical basis for determining their balanced fiber winding angle.

## 2. Analysis Procedure

**Figure 2:**
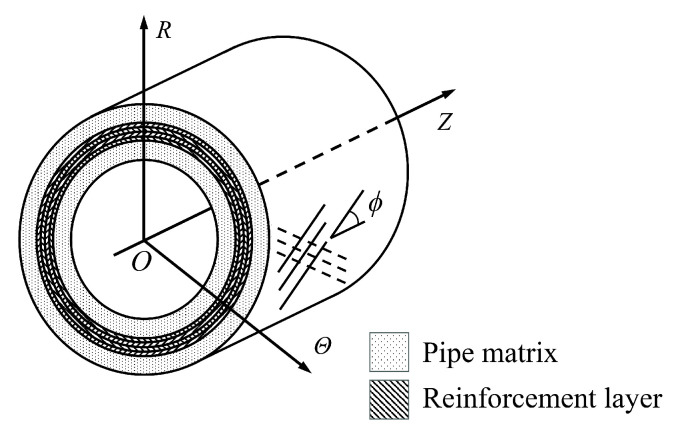
The theoretical model of thick-walled FRF pipes.

Based on the finite deformation theory, Figure 2 illustrates a cylindrical coordinate system defined in the reference configuration, where *R*, Θ, and *Z* are the radial, circumferential, and axial coordinates, respectively. It should be noted that the selection of the reference configuration is primarily justified by the fact that the constitutive relation of the polymer matrix is typically defined with respect to the stress-free state. Furthermore, during numerical analysis, performing numerical integration over a fixed initial geometry avoids the complexity associated with tracking the deformed mesh.

### 2.1. Fundamental Assumptions

To capture the primary mechanical behavior of the pipe while ensuring the solvability of the established model, the following assumptions are introduced to address secondary influencing factors:

1. The pipe matrix is modeled as a homogeneous, continuous, and isotropic hyperelastic material, while the reinforcement layer is treated as a homogeneous, continuous, and anisotropic linear elastic material. Both materials are assumed to be free of initial defects and remain undamaged and failure-free within the pressure and deformation ranges considered in the analysis;
2. After the manufacturing process, the pipe matrix and the reinforcement layer are perfectly bonded at the interface and do not experience macroscopic relative sliding or delamination under external loading;
3. The pipe is in a stress-free state after fabrication;
4. The pressure medium is modeled as an ideal pressure transmission medium, and its compressibility, density, and other relevant properties do not affect the balanced performance of the pipe.

### 2.2. Displacement and Strain Analysis

When a thick-walled FRF pipe deforms under internal pressure, the displacement field *u* of any material point in the continuum can be defined as:

(1) u=x−X

where X=(R,Θ,Z) represents the coordinates of a material point in the reference configuration, and x=(r,θ,z) represents the coordinates of the same material point in the current configuration. Therefore, the displacement field can be expressed as a function that depends only on the coordinates of the reference configuration:

(2) u=u(X)

When the unit vectors in the radial, circumferential and axial directions of the cylindrical coordinate system in the reference configuration are denoted by $e_{R}$, $e_{\Theta }$ and $e_{Z}$, respectively, the displacement field can be expressed as:

(3) $$u=u_{R}e_{R}+u_{\Theta }e_{\Theta }+u_{Z}e_{Z}$$

where $u_{R}$, $u_{\Theta }$, and $u_{Z}$ are the components of the displacement vector in the radial, circumferential, and axial directions, respectively. Due to the symmetry of the pipe’s geometry and the internal pressure loading, all field quantities are independent of the circumferential coordinate. Based on this symmetry, the displacement field can be simplified.

For a thick-walled FRF pipe, the radial displacement needs to account for the nonlinear and complex deformation along the thickness direction induced by end constraints:

(4) $$u_{R}=u_{R}\left(R,Z\right)$$

Under geometric and loading conditions with axial symmetry, if the pipe material is isotropic, circumferential deformation is usually not induced. However, the orthotropic anisotropy of the reinforcement layer may lead to circumferential deformation. Nevertheless, the symmetric layup pattern adopted in this study (with fiber winding angles of ±ϕ) and the axial symmetry condition jointly suppress such deformation. Meanwhile, to reduce model complexity, the circumferential displacement is assumed to be zero:

(5) $$u_{\Theta }=0$$

For thick-walled FRF pipes with an axial length comparable to the radius scale, the end constraint effect is significant, leading to a non-uniform distribution of axial displacement along the pipe. Meanwhile, if the pipe matrix is a polymer exhibiting approximately incompressible behavior, such as rubber, the radial and axial strains are coupled. Therefore, the axial displacement is given by:

(6) $$u_{Z}=u_{Z}\left(R,Z\right)$$

Based on the above displacement field, the deformation gradient *F* can be defined as:

(7) $$F=\frac{\partial (X+u)}{\partial X}=I+\nabla u$$

where ∇u denotes the displacement gradient tensor, and *I* represents the identity tensor. The component form of the displacement gradient is given by:

(8) $$\nabla u=\left[\begin{array}{ccc} \frac{\partial u_{R}}{\partial R} & 0 & \frac{\partial u_{R}}{\partial Z}\\ 0 & \frac{u_{R}}{R} & 0\\ \frac{\partial u_{Z}}{\partial R} & 0 & \frac{\partial u_{Z}}{\partial Z} \end{array}\right]$$

In the Lagrangian description, the Green–Lagrange strain *E* can be defined by the deformation gradient as:

(9) $$E=\frac{1}{2}\left(F^{T}F-I\right)$$

The matrix form of *E* in the cylindrical coordinate system is given by:

(10) $$E=\left[\begin{array}{ccc} E_{RR} & 0 & E_{RZ}\\ 0 & E_{\Theta \Theta } & 0\\ E_{RZ} & 0 & E_{ZZ} \end{array}\right]$$

The expressions of each non-zero component in the above equation are given by:

(11) $$\begin{array}{c} E_{RR}=\frac{\partial u_{R}}{\partial R}+\frac{1}{2}\left[{\left(\frac{\partial u_{R}}{\partial R}\right)}^{2}+{\left(\frac{\partial u_{Z}}{\partial R}\right)}^{2}\right]\\ E_{\Theta \Theta }=\frac{u_{R}}{R}+\frac{1}{2}{\left(\frac{u_{R}}{R}\right)}^{2}\\ E_{ZZ}=\frac{\partial u_{Z}}{\partial Z}+\frac{1}{2}\left[{\left(\frac{\partial u_{R}}{\partial Z}\right)}^{2}+{\left(\frac{\partial u_{Z}}{\partial Z}\right)}^{2}\right]\\ E_{RZ}=\frac{1}{2}\left(\frac{\partial u_{R}}{\partial Z}+\frac{\partial u_{Z}}{\partial R}+\frac{\partial u_{R}}{\partial R}\frac{\partial u_{R}}{\partial Z}+\frac{\partial u_{Z}}{\partial R}\frac{\partial u_{Z}}{\partial Z}\right) \end{array}$$

### 2.3. Constitutive Relations

For polymers, the hyperelastic constitutive relationship can be characterized by calibrating the strain energy density function $\Psi_{r}$ [30]. The second Piola–Kirchhoff stress $S_{r}$ is obtained as:

(12) $$S_{r}=\frac{\partial \Psi_{r}}{\partial E}$$

Considering that the tensile modulus of fibers in the reinforcement layer is usually much higher than that of the surrounding matrix material, the matrix material within the layer is simplified from a hyperelastic model to a linear elastic model.

**Figure 3:**
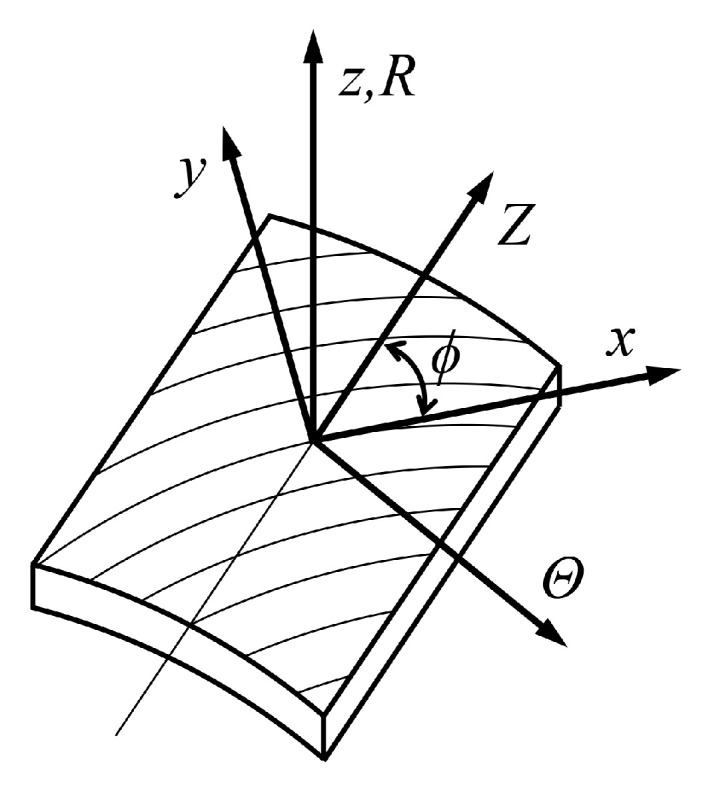
Relationship between the material principal coordinate system and the cylindrical coordinate system of the reinforcement layer.

In the material principal coordinate system shown in Figure 3 (where the *x* axis aligns with the fiber direction, the *y* axis represents the in-plane transverse direction, and the *z* axis denotes the thickness direction), fibers are oriented along the *x* direction, while the mechanical response in the *y* and *z* directions is dominated by the linear elastic matrix phase. Given this material property distinction, the reinforcement layer exhibits transverse isotropy in the *y*-*z* plane, and its engineering constants satisfy the following symmetry conditions:

(13) $$\begin{array}{c} E_{rfy}=E_{rfz}\\ G_{rfxy}=G_{rfxz}\\ \nu_{rfyx}=\nu_{rfzx} \end{array}$$

where $E_{rfy}$ and $E_{rfz}$ are the transverse elastic moduli, $G_{rfxy}$ and $G_{rfxz}$ are the longitudinal-transverse shear moduli, and $\nu_{rfyx}$ and $\nu_{rfzx}$ are the longitudinal-transverse Poisson’s ratios.

Furthermore, by virtue of the transverse isotropy in the *y*-*z* plane, the shear modulus $G_{rfyz}$ is:

(14) $$G_{rfyz}=\frac{E_{rfy}}{2(1+\nu_{rfyz})}$$

**Figure 4:**
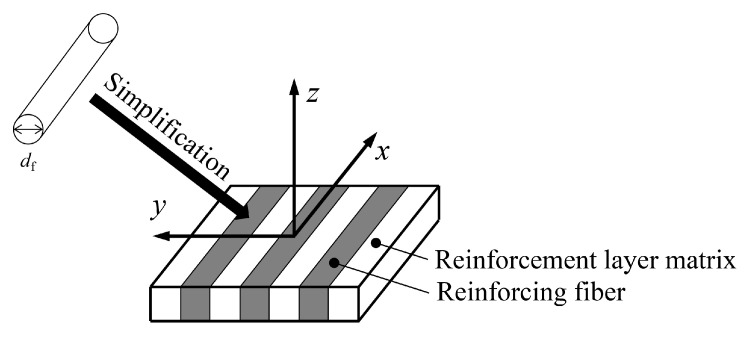
Simplified model of the reinforcement layer.

The simplified model of the reinforcement layer is depicted in Figure 4. If the diameter and center-to-center distance of reinforcing fibers are denoted by $d_{f}$ and $a_{f}$, then the volume fraction of the reinforcing fiber $V_{f}$ and the volume fraction of the matrix $V_{r}$ are:

(15) $$\begin{array}{c} V_{f}=\frac{\pi {d_{f}}^{2}}{4{a_{f}}^{2}}\\ V_{r}=1-V_{f} \end{array}$$

The elastic modulus $E_{rfx}$ of the reinforcement layer in the *x* direction and the corresponding Poisson’s ratio $\nu_{rfyx}$ are given by:

(16) $$\begin{array}{c} E_{rfx}=V_{f}E_{f}+V_{r}E_{r}\\ \nu_{rfyx}=V_{f}\nu_{f}+V_{r}\nu_{r} \end{array}$$

where $E_{f}$ and $E_{r}$ represent the elastic moduli of the fiber and matrix, respectively, and $\nu_{f}$ and $\nu_{r}$ represent the Poisson’s ratios of the fiber and the matrix.

The $G_{rfxy}$ is calculated using the Halpin–Tsai equation [20]:

(17) $$G_{rfxy}=G_{r}\frac{1+\xi \eta V_{f}}{1-\eta V_{f}}$$

where $G_{r}$ represents the shear modulus of the matrix and ξ is the shape factor. The intermediate variable η is defined as:

(18) $$\eta =\frac{\frac{G_{f}}{G_{r}}-1}{\frac{G_{f}}{G_{r}}+\xi }$$

where $G_{f}$ represents the shear modulus of the reinforcing fiber.

In the *y*-*z* plane, the transverse deformation behavior of the reinforcement layer is described by a series coupling model comprising the fiber and matrix within the layer. Therefore, the Poisson’s ratio $\nu_{rfyz}$ is:

(19) $$\nu_{rfyz}=\frac{\nu_{f}\nu_{r}}{V_{f}\nu_{r}+V_{r}\nu_{f}}$$

The transverse elastic modulus $E_{rfy}$ of the reinforcement layer is also determined through the Halpin–Tsai equation:

(20) $$E_{rfy}=E_{r}\frac{1+\xi^{\prime }\eta^{\prime }V_{f}}{1-\eta^{\prime }V_{f}}$$

where $\xi^{\prime }$ denotes the shape factor corresponding to the transverse elastic modulus. The intermediate variable $\eta^{\prime }$ is defined as:

(21) $$\eta^{\prime }=\frac{\frac{E_{f}}{E_{r}}-1}{\frac{E_{f}}{E_{r}}+\xi^{\prime }}$$

The stiffness matrix of the reinforcement layer in its material principal coordinate system can be obtained by inverting the compliance matrix. The normal stiffness matrix is:

(22) $$\left[\begin{array}{ccc} C_{xx} & C_{xy} & C_{xz}\\ C_{xy} & C_{yy} & C_{yz}\\ C_{xz} & C_{yz} & C_{zz} \end{array}\right]={\left[\begin{array}{ccc} \frac{1}{E_{rfx}} & -\frac{\nu_{rfyx}}{E_{rfx}} & -\frac{\nu_{rfyx}}{E_{rfx}}\\ -\frac{\nu_{rfyx}}{E_{rfx}} & \frac{1}{E_{rfy}} & -\frac{\nu_{rfyz}}{E_{rfy}}\\ -\frac{\nu_{rfyx}}{E_{rfx}} & -\frac{\nu_{rfyz}}{E_{rfy}} & \frac{1}{E_{rfy}} \end{array}\right]}^{-1}$$

Including the independent shear terms, the stiffness vector ${\bar{C}}_{rf}^{mat}$ of the reinforcement layer in the material principal coordinate system is:

(23) $${\bar{C}}_{rf}^{mat}={\left[C_{xx},C_{yy},C_{zz},C_{xy},C_{xz},C_{yz},G_{yz},G_{xz},G_{xy}\right]}^{T}$$

where $G_{yz}$, $G_{xz}$ and $G_{xy}$ denote the shear moduli $G_{rfyz}$, $G_{rfxz}$ and $G_{rfxy}$ defined previously.

In the cylindrical coordinate system, the stiffness matrix of the reinforcement layer has the following form:

(24) $$C_{rf}^{cyl}=\left[\begin{array}{cccccc} C_{11} & C_{12} & C_{13} & C_{14} & 0 & 0\\ C_{12} & C_{22} & C_{23} & C_{24} & 0 & 0\\ C_{13} & C_{23} & C_{33} & C_{34} & 0 & 0\\ C_{14} & C_{24} & C_{34} & C_{44} & 0 & 0\\ 0 & 0 & 0 & 0 & C_{55} & C_{56}\\ 0 & 0 & 0 & 0 & C_{56} & C_{66} \end{array}\right]$$

This stiffness matrix can also be represented by a vector composed of its independent components:

(25) $${\bar{C}}_{rf}^{cyl}={\left[C_{11},C_{12},C_{13},C_{14},C_{22},C_{23},C_{24},C_{33},C_{34},C_{44},C_{55},C_{56},C_{66}\right]}^{T}$$

The stiffness components in the material principal coordinate system and the cylindrical coordinate system are related through the transformation matrix *A*:

(26) $${\bar{C}}_{rf}^{cyl}=A{\bar{C}}_{rf}^{mat}$$

According to Figure 3, the coordinate transformation direction cosine matrix *T* can be defined as:

(27) $$T=\left[\begin{array}{ccc} 0 & 0 & 1\\ n & -m & 0\\ m & n & 0 \end{array}\right]$$

where *m* and *n* denote the cosine and sine of the fiber winding angle. Therefore, *A* can be expressed as:

(28) $$A=\left[\begin{array}{ccccccccc} 0 & 0 & 1 & 0 & 0 & 0 & 0 & 0 & 0\\ 0 & 0 & 0 & 0 & n^{2} & m^{2} & 0 & 0 & 0\\ 0 & 0 & 0 & 0 & m^{2} & n^{2} & 0 & 0 & 0\\ 0 & 0 & 0 & 0 & mn & -mn & 0 & 0 & 0\\ n^{4} & m^{4} & 0 & 2m^{2}n^{2} & 0 & 0 & 0 & 0 & 4m^{2}n^{2}\\ m^{2}n^{2} & m^{2}n^{2} & 0 & m^{4}+n^{4} & 0 & 0 & 0 & 0 & -4m^{2}n^{2}\\ mn^{3} & -m^{3}n & 0 & m^{3}n-mn^{3} & 0 & 0 & 0 & 0 & 2m^{3}n-2mn^{3}\\ m^{4} & n^{4} & 0 & 2m^{2}n^{2} & 0 & 0 & 0 & 0 & 4m^{2}n^{2}\\ nm^{3} & -mn^{3} & 0 & -m^{3}n+mn^{3} & 0 & 0 & 0 & 0 & -2m^{3}n+2mn^{3}\\ m^{2}n^{2} & m^{2}n^{2} & 0 & -2m^{2}n^{2} & 0 & 0 & 0 & 0 & {\left(m^{2}-n^{2}\right)}^{2}\\ 0 & 0 & 0 & 0 & 0 & 0 & n^{2} & m^{2} & 0\\ 0 & 0 & 0 & 0 & 0 & 0 & -mn & mn & 0\\ 0 & 0 & 0 & 0 & 0 & 0 & m^{2} & n^{2} & 0 \end{array}\right]$$

The strain energy density function of the reinforcement layer $\Psi_{rf}$ can be expressed as:

(29) $$\Psi_{rf}=\frac{1}{2}{\bar{E}}^{T}C_{rf}^{cyl}\bar{E}$$

where $\bar{E}={\left[E_{RR},E_{\Theta \Theta },E_{ZZ},2E_{\Theta Z},2E_{RZ},2E_{R\Theta }\right]}^{T}$. The second Piola–Kirchhoff stress of the reinforcement layer $S_{rf}$ is:

(30) $$S_{rf}=\frac{\partial \Psi_{rf}}{\partial E}$$

### 2.4. Governing Equation

Since the constitutive relations of the pipe matrix and the reinforcement layer are both characterized by the strain energy density function, the governing equation is established based on the principle of minimum potential energy. The total potential energy Π(u) of a thick-walled FRF pipe is:

(31) Π(u)=U(u)−W(u)

where U(u) and W(u) represent the strain energy and the work done by the internal pressure, respectively. For a laminated pipe, the total strain energy is:

(32) $$U\left(u\right)=\sum_{k=1}^{N_{l}}U^{\left(k\right)}\left(u\right)$$

where $N_{l}$ represents the total number of layers. In the cylindrical coordinate system, the strain energy of the *k*-th layer, denoted by $U^{\left(k\right)}$, can be expressed as:

(33) $$U^{\left(k\right)}\left(u\right)=2\pi \int \int \Psi^{\left(k\right)}RdRdZ$$

Internal pressure *p* acts perpendicularly on the inner surface of the pipe and the inner surfaces of the end plates. Therefore, the total work done by external forces is:

(34) $$W\left(u\right)=W_{inner}\left(u\right)+W_{end}\left(u\right)$$

where $W_{inner}$ and $W_{end}$ are the external work performed on the inner cylindrical surface and end plates, respectively. Assuming that the direction of the internal pressure remains perpendicular to the loaded surfaces, the work expressions are given by:

(35) $$\begin{array}{c} W_{inner}=2\pi pR_{in}\int_{0}^{L}u_{R}\left(R_{in},Z\right)dZ\\ W_{end}=2\pi p\int_{0}^{R_{in}}u_{Z}\left(R,L\right)RdR \end{array}$$

where $R_{in}$ represents the inner radius of the pipe and *L* represents its axial length.

The actual displacement field renders the total potential energy functional stationary in the sense of calculus of variations, i.e., it satisfies the first-order variation condition:

(36) $$\delta \Pi \left(u\right)=\delta U\left(u\right)-\delta W\left(u\right)=0$$

By performing variational operations on Equations (32) and (34), the governing equation in the reference configuration can be obtained:

(37) $$\nabla \;\cdot \;\left(F\;\cdot \;S\right)=0$$

where ∇ is the divergence operator.

Substituting the geometric relations derived from the displacement field and the constitutive relations obtained from the material model into Equation (37), a system of nonlinear partial differential equations with the displacement components $u_{R}\left(R,Z\right)$ and $u_{Z}\left(R,Z\right)$ can be obtained. The nonlinearity of the equation stems from the strain–displacement relationship containing higher-order terms of the displacement gradient and the nonlinear stress–strain response of the material. Meanwhile, the boundary conditions of the multi-layer pipe are rather complex, making it infeasible to obtain an analytical solution.

To transform this continuum mechanics boundary value problem into a numerically tractable form, the Rayleigh–Ritz method is applied based on the principle of minimum potential energy. Displacement trial functions satisfying the geometric boundary conditions are constructed to approximate the actual displacement field.

### 2.5. Numerical Solution

For a pipe with one end fixed (Z=0), the displacement is fully restrained:

(38) $$\begin{array}{c} u_{R}\left(R,0\right)=0\\ u_{Z}\left(R,0\right)=0 \end{array}$$

At the free end (Z=L), the rigid end plate fully constrains the radial displacement while permitting free axial deformation:

(39) $$\begin{array}{c} u_{R}\left(R,L\right)=0\\ u_{Z}\left(R,L\right)=Q\left(L\right) \end{array}$$

where $Q\left(L\right)$ represents the axial deformation at the free end.

Based on the above displacement boundary conditions, the radial and axial displacement trial functions are assumed to take the following separable-variable functional form:

(40) $$\begin{array}{c} u_{R}\left(R,Z\right)=P\left(R\right)f\left(Z\right)\\ u_{Z}\left(R,Z\right)=Q\left(Z\right)+G\left(R\right)g\left(Z\right) \end{array}$$

where $f\left(Z\right)$ and $g\left(Z\right)$ are geometric boundary-compatible functions designed to ensure the trial functions automatically satisfy the geometric boundary conditions. $P\left(R\right)$, $Q\left(Z\right)$ and $G\left(R\right)$ are spatial shape functions describing the spatial distribution of the displacement field.

According to Equations (38) and (39), $f\left(Z\right)$ and $G\left(Z\right)$ are defined as dimensionless power functions:

(41) $$\begin{array}{c} f\left(Z\right)={\left(\frac{Z}{L}\right)}^{m_{f}}{\left(1-\frac{Z}{L}\right)}^{n_{f}}\\ g\left(Z\right)={\left(\frac{Z}{L}\right)}^{m_{g}}{\left(1-\frac{Z}{L}\right)}^{n_{g}} \end{array}$$

where $m_{f}$, $n_{f}$, $m_{g}$ and $n_{g}$ are all positive integers.

Based on Weierstrass approximation theorem [31], any continuous function on a closed interval can be uniformly approximated by polynomial functions to arbitrary precision. Thus, when shape functions are expressed as polynomial expansions, theoretically, by adjusting the undetermined coefficients, all possible deformation characteristics of the thick-walled FRF pipe under maximum working internal pressure can be characterized. Shape functions are expressed as dimensionless power series polynomials:

(42) $$\begin{array}{c} P\left(R\right)=\sum_{p=1}^{N_{p}}a_{p}{\left(\frac{R}{R_{in}}\right)}^{n_{p}}\\ Q\left(Z\right)=\sum_{q=1}^{N_{q}}b_{q}{\left(\frac{Z}{L}\right)}^{n_{q}}\\ G\left(R\right)=\sum_{g=1}^{N_{g}}c_{g}{\left(\frac{R}{R_{in}}\right)}^{n_{g}} \end{array}$$

where $a_{p}$, $b_{q}$ and $c_{g}$ are all undetermined coefficients. $n_{p}$, $n_{q}$ and $n_{g}$ are all non-negative integers. $N_{p}$, $N_{q}$ and $N_{g}$ are the truncation orders of the polynomial series.

It should be noted that the above boundary condition functions and shape functions are rendered dimensionless by normalizing coordinates with their respective characteristic lengths. This formulation ensures dimensional consistency in the total potential energy expression, effectively avoiding numerical ill-conditioning arising from significant discrepancies in physical dimensions, thereby enhancing the stability and convergence of the subsequent numerical solutions. Furthermore, the use of dimensionless forms facilitates the generalization of the findings of this work to geometrically similar pipes of different scales.

Under axisymmetric conditions, the integrand is independent of the circumferential coordinate, and the corresponding volume integral and surface integral can be reduced to two-dimensional numerical integration. Therefore, Equation (33) can be expressed as:

(43) $$U^{\left(k\right)}\left(u\right)=2\pi \sum_{i=1}^{n_{R}}\sum_{j=1}^{n_{Z}}w_{i}w_{j}\Psi^{\left(k\right)}\left(R_{i},Z_{j}\right)R_{i}$$

where $w_{i}$ and $w_{j}$ are the integration weights in the radial and axial directions, respectively. $\left(R_{i},Z_{j}\right)$ are the coordinates of the Gaussian quadrature sampling points. $n_{R}$ and $n_{Z}$ represent the number of integration points in each direction. Equation (35) can be expressed in discrete form as:

(44) $$\begin{array}{c} W_{inner}=2\pi pR_{in}\sum_{j=1}^{n_{Z}}w_{j}u_{R}\left(R_{in},Z_{j}\right)\\ W_{end}=2\pi p\sum_{i=1}^{n_{R}}w_{i}u_{Z}\left(R_{i},L\right)R_{i} \end{array}$$

The total potential is thus expressed as a multivariate function of the undetermined coefficients, where the coefficient vector $\bar{d}$ is:

(45) $$\bar{d}={\left[a_{1},a_{2},\ldots ,a_{p},b_{1},b_{2},\ldots ,b_{q},c_{1},c_{2},\ldots ,c_{g}\right]}^{T}$$

According to the Rayleigh–Ritz method, a system of nonlinear algebraic equations regarding the undetermined coefficients can be obtained:

(46) $$\frac{\partial \Pi }{\partial d_{i}}=0$$

where $i=1,2,\ldots ,(N_{p}+N_{q}+N_{g})$. The equations are solved iteratively using the Newton–Raphson method:

(47) $$d^{\left(m+1\right)}=d^{\left(m\right)}-H^{-1}Res$$

where *m* represents the iteration number. Res and *H* are the residual vector and the Jacobian matrix, respectively, with components given by:

(48) $$\begin{array}{c} Res_{i}=\frac{\partial \Pi }{\partial d_{i}}\\ H_{ij}=\frac{\partial^{2}\Pi }{\partial d_{i}\partial d_{j}} \end{array}$$

The partial derivatives in the above equations can be computed using the central difference method:

(49) $$\begin{array}{c} \frac{\partial \Pi }{\partial d_{i}}=\frac{\Pi \left(d+he_{i}\right)-\Pi \left(d-he_{i}\right)}{2h}\\ \frac{\partial^{2}\Pi }{\partial d_{i}\partial d_{j}}=\frac{\Pi \left(d+he_{i}+he_{j}\right)-\Pi \left(d+he_{i}\right)-\Pi \left(d+he_{j}\right)+\Pi \left(d\right)}{h^{2}} \end{array}$$

where *h* is the difference step size, and $e_{i}$, $e_{j}$ are unit basis vectors corresponding to the *i*-th and *j*-th directions of the coefficient vector, respectively.

To ensure that the displacement trial function can approximate the actual displacement field, convergence during numerical iteration is declared when the following three convergence criteria are simultaneously satisfied:

(50) $$\begin{array}{c} {∥{Res}^{\left(m\right)}∥}_{2}<tol\\ {∥d^{\left(m+1\right)}-d^{\left(m\right)}∥}_{2}<tol\\ \frac{\left|\Pi^{\left(m+1\right)}-\Pi^{\left(m\right)}\right|}{\left|\Pi^{\left(m\right)}\right|}<tol \end{array}$$

where tol is the convergence tolerance.

## 3. Results and Discussion

This section first clarifies the benchmarks used to verify the reliability of the semi-analytical method, then analyzes the completeness of the displacement trial function and the robustness of the Newton–Raphson solution method. By comparing the calculation results of the semi-analytical method with the verification benchmarks, the deformation mechanism of the thick-walled FRF pipe is analyzed, and the influence of the fiber winding angle on its balanced performance is discussed.

### 3.1. Verification Benchmarks

**Figure 5:**
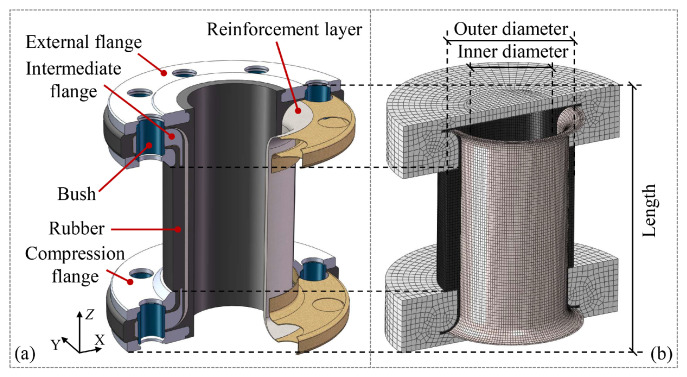
The aramid fiber-reinforced thick-walled rubber pipe: (**a**) Geometric structure. (**b**) Finite element model.

To verify the reliability of the semi-analytical method proposed in this work, the finite element model and its corresponding experimental results from Reference [32] are adopted as the benchmark for validation. In that study, the research object is an aramid fiber-reinforced rubber thick-walled pipe with a three-flange structure, as illustrated in Figure 5a. The manufacturing parameters of the pipe are shown in Table 1. In the finite element modeling, the rubber matrix and reinforcement layers are simulated using C3D8R and M3D4R elements, respectively, and the embedded element technique is used to embed the reinforcement layer into the rubber matrix. The boundary conditions of the model are set as one end fixed and the other end free to deform axially, and the internal pressure is applied as a uniformly distributed load on the inner surfaces of the pipe and end plates.

The finite element model, as shown in Figure 5b, is consistent with the finite element model in the above-mentioned literature. It should be noted that the geometric simplification adopted in the referenced finite element model omits the region where the reinforcement layer wraps around the intermediate flange and is bolted in place, as shown in the yellow part of Figure 5a. The structural dimensions and boundary conditions used in the semi-analytical method in this paper are the same as those of the finite element model, but only the straight section of the pipe (length of 88.8 mm) is modeled.

In terms of material modeling, the semi-analytical method treats the rubber matrix within the reinforcement layer as a linear elastic material. The corresponding elastic parameters are derived from the Neo-Hookean hyperelastic model calibrated using experiments from Reference [32]:

(51) $$\Psi_{r}=C_{10}\left({\bar{I}}_{1}-3\right)+\frac{1}{D_{1}}{\left(J-1\right)}^{2}$$

where $C_{10}=1.98$ is the parameter governing the shear response of the material, and $D_{1}=8.62\times 10^{-3}$ controls its volumetric compressibility. Here, ${\bar{I}}_{1}$ and *J* denote the first deviatoric invariant and the determinant of the deformation gradient (i.e., the volume change ratio), respectively.

Based on this hyperelastic model, $G_{r}$ and $K_{r}$ required for linear elastic approximation can be determined as:

(52) $$\begin{array}{c} G_{r}=2C_{10}\\ K_{r}=2/D_{1}\; \end{array}$$

From these, $\nu_{r}$ and $E_{r}$ can then be calculated:

(53) $$\begin{array}{c} \nu_{r}=\frac{3K_{r}-2G_{r}}{2\left(3K_{r}+G_{r}\right)}\\ E_{r}=2G_{r}\left(1+\nu_{r}\right) \end{array}$$

### 3.2. Selection and Verification of Displacement Trial Functions

The core of the semi-analytical method lies in approximating the displacement field through predefined displacement trial functions. Consequently, the completeness of displacement trial functions and the robustness of the Newton–Raphson method solution process are two key factors ensuring the reliability of the method.

To determine the optimal number of undetermined coefficients that ensures both accuracy and computational efficiency of displacement trial functions, an extended series model is constructed by incrementally increasing the number of coefficients. If the axial deformation quantity tends to stabilize and the overall deformation behavior converges, then the corresponding displacement trial functions are deemed complete. Based on Equation (40), displacement trial functions with different parameter quantities are constructed, as shown in Table 2. The primary distinction among these functions lies in the degree to which higher-order terms and coupling terms between radial and axial coordinates are retained.

The axial deformation results computed using these different trial functions are presented in Table 3. It can be observed that when the number of coefficients increases from six to seven, the axial deformation shifts from positive to negative, indicating that the trial functions have captured the dominant mechanism responsible for axial contraction of the pipe. As the number of coefficients further increases to nine and ten, the axial deformation stabilizes at −0.38 mm, confirming convergence of the solution.

**Figure 6:**
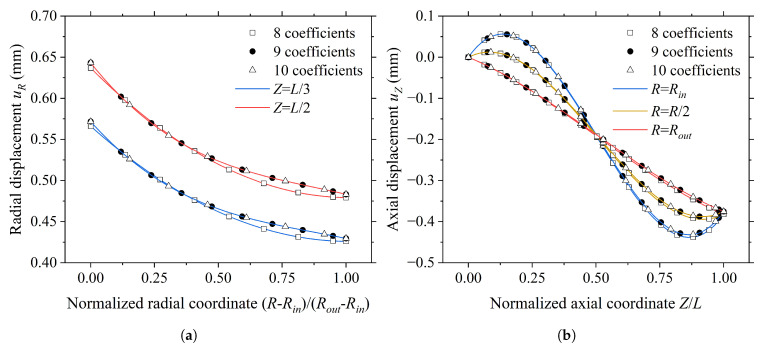
Displacement curves computed using different displacement trial functions: (**a**) Radial displacement. (**b**) Axial displacement.

To further verify the completeness of the displacement trial function, Figure 6 compares the axial and radial displacement fields computed using the eight-, nine-, and ten-coefficient functions. The results show that the displacement curves obtained from the nine- and ten-coefficient functions nearly overlap completely along the key path, while the deviation of the eight-coefficient functions relative to the latter two is negligible. This indicates that the numerical solution of the displacement field has fully converged when the number of coefficients exceeds eight. Additionally, as shown in Table 4, the eight-coefficient function maintains a comparable level of accuracy while offering improved computational efficiency.

**Figure 7:**
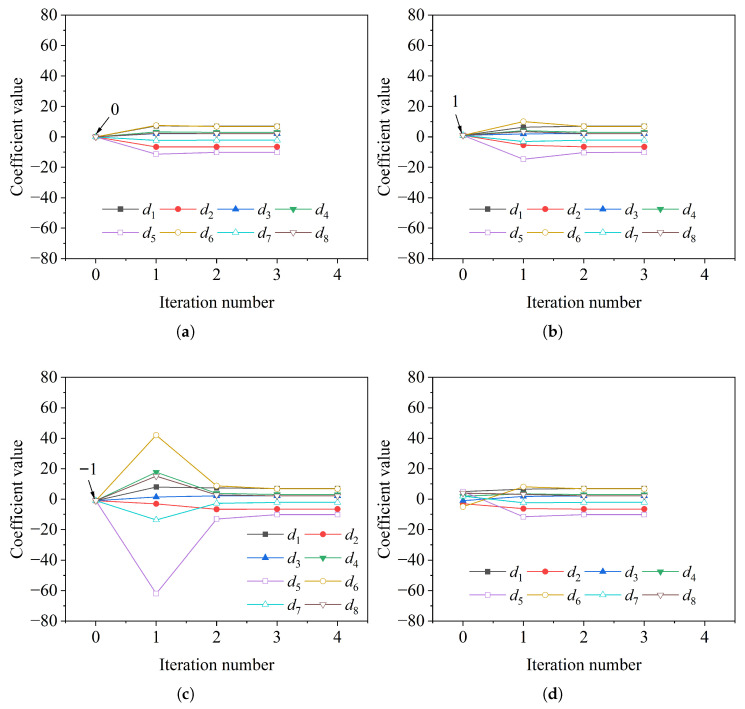
Variation of the initial coefficient values with respect to the number of iterations: (**a**) Zero vector. (**b**) All-positive vector. (**c**) All-negative vector. (**d**) Random vector.

Since the numerical solution obtained by the Newton–Raphson method can be sensitive to the initial guess, multiple initial coefficient vectors are tested for the eight-coefficient displacement trial function, including the zero vector, an all-positive vector, an all-negative vector, and a random vector. The iterative convergence process is shown in Figure 7. Despite significant differences in the initial values, all cases converge rapidly within three to four iterations, yielding identical final values of the undetermined coefficients. These converged coefficient values are listed in Table 5. Notably, the coefficients already approach their final convergence values after the first or second iteration, demonstrating a high initial convergence rate. The results indicate that the potential energy functional, constructed based on the principle of minimum potential energy, exhibits convexity, and the numerical iteration process is insensitive to the choice of initial guess.

### 3.3. Deformation Mechanism

When the reinforcing fiber is wound at the experience-based engineering balanced angle of $\pm 53.1^{{}^{\circ}}$, the axial deformation calculated by the semi-analytical method is −0.38 mm, as shown in Table 3. This value is below the commonly adopted engineering tolerance standard of ±1 mm, indicating effective suppression of axial deformation in the pipe under maximum working pressure and confirming its self-balanced state.

To validate the reliability of the semi-analytical method’s predictions, results are compared against experimental data reported in Reference [32]. The measured axial deformations of the four thick-walled FRF pipe specimens are −0.05 mm, 0.25 mm, 0.35 mm, and −0.05 mm, with an average value of 0.125 mm. The scattered distribution and average value of the experimental results both fall within the engineering tolerance standard, indicating that all specimens still satisfy the self-balancing requirement under actual process fluctuations. Although the prediction from the semi-analytical method differs from the experimental mean, it shares the same sign as the measured value (−0.05 mm) and is close in absolute value to the other measured value (+0.35 mm). Meanwhile, the prediction satisfies the balancing evaluation criterion that the absolute value of axial deformation is less than 1 mm. This demonstrates that the method can reliably predict the macroscopic balancing performance indicators and validates the engineering applicability of the selected eight-coefficient displacement trial function.

To further examine the accuracy of the deformation mechanism revealed by the semi-analytical method, the displacement field contour plots obtained from the semi-analytical method are compared with those computed using the finite element method. The deformation contour plots of the thick-walled FRF pipe under identical loading conditions are generated through the finite element modeling procedure described in Reference [32].

**Figure 8:**
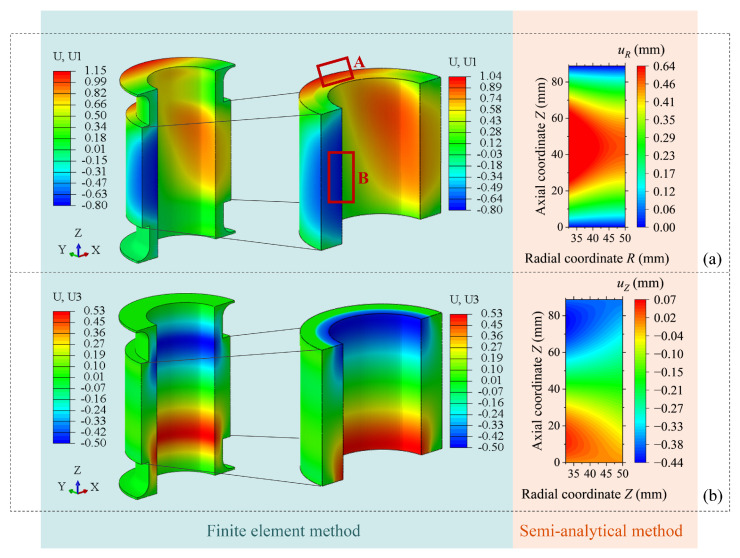
Displacement contour plots obtained using the semi-analytical method and the finite element method. (**a**) Radial deformation. (**b**) Axial deformation.

By comparing the displacement fields calculated by the two methods, as shown in Figure 8, it can be seen that for the radial displacement, the radial displacement range of the straight pipe section in the finite element model (with flange section) is −0.8 mm to 1.04 mm, with the maximum value located in Region A on the upper surface of the straight pipe section and the minimum value in Region B at the axial middle of the pipe inner surface. The range obtained by the semi-analytical method is 0 mm to 0.64 mm, with the maximum value appearing at the axial middle of the inner surface and the minimum value at the fixed end and free end. For the axial displacement, although the contour distribution trends obtained by the two methods are generally consistent, there are still certain differences in the calculated values.

**Figure 9:**
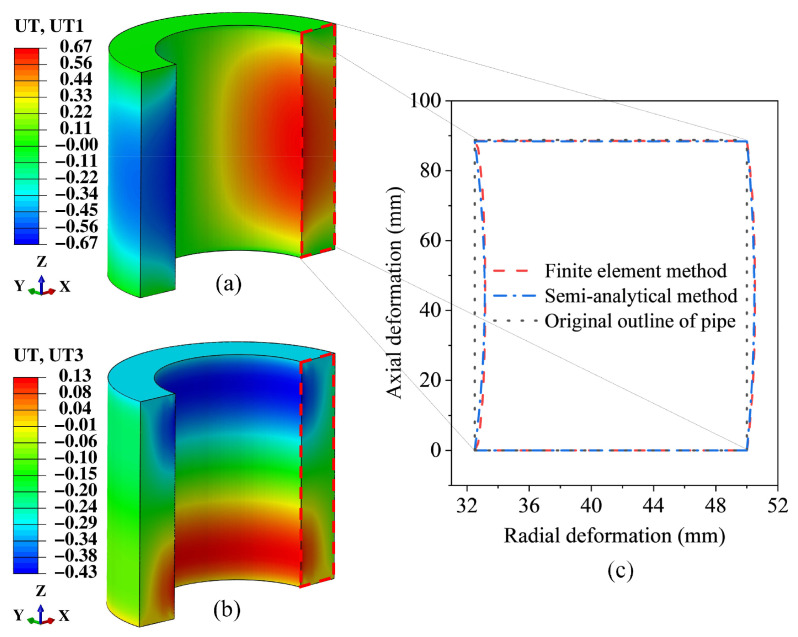
Calculation results of the finite element method and semi-analytical method for the straight pipe section: (**a**) Radial displacement by the finite element method. (**b**) Axial displacement by the finite element method. (**c**) Outer contour displacement of the pipe calculated by the two methods.

To clarify the source of the above differences, a finite element model containing only the straight pipe section is established, and its calculation results are shown in Figure 9a,b. In addition, Figure 9c extracts and compares the outer contour deformation of the pipe predicted by the semi-analytical method and the finite element method for the straight pipe section. The results show that under the same boundary conditions and loads, the two methods have minor differences in the overall distribution of the predicted displacement fields of the straight pipe section. This indicates that the discrepancies observed in Figure 8 are mainly caused by the local constraint effect of the flange connection region.

Furthermore, the overall axial deformation of the pipe obtained by the finite element model of the straight pipe section is −0.3 mm, which differs by 0.08 mm from the result of the semi-analytical method (−0.38 mm). The difference is small and both are within the engineering allowable range of ±1 mm, indicating that the semi-analytical method can reliably predict the balanced performance of the thick-walled FRF pipe.

Compared with the finite element method, the semi-analytical method proposed in this work expresses the displacement field as an explicit function, which parameterizes the deformation distribution of the thick-walled pipe and enables a direct mapping between mathematical parameters and physical mechanisms. Based on the eight-coefficient displacement trial function, the deformation mechanism of the thick-walled FRF pipe in the balanced state can be clearly understood.

The eight coefficients, as shown in Table 5, quantitatively characterize the dominant deformations shown in Figure 8 and Figure 9. For radial deformation, coefficient $d_{1}>0$ represents the overall expansion trend caused by internal pressure. Coefficient $d_{2}$ with opposite sign and similar absolute value quantifies the rapid constraint effect of the reinforcement layer as the radius increases, revealing the physical transition from matrix-dominated expansion near the inner wall to fiber-dominated anti-expansion within the reinforcement layer region. The relatively small magnitude of $d_{3}$ results in a smoother displacement profile in the outer wall region, reflects both the continuity of the outermost pipe layer and its gradient-smoothing effect on the radial deformation. Collectively, these three coefficients constitute a quadratic function about the radial coordinate, and physically lead to the “inner expansion and outer suppression” non-uniform radial deformation gradient.

For axial deformation, coefficients $d_{4}$, $d_{5}$ and $d_{6}$ jointly determine the axial displacement distribution along the pipe length, with their alternating signs indicating a complex nonlinear deformation pattern rather than simple uniform elongation or shortening. The most significant coefficients, $d_{7}$ and $d_{8}$, exhibit equal magnitudes but opposite signs and together represent a locally warped displacement distribution that is antisymmetric about the mid-section of the pipe, induced by the rigid constraint at the end flange. Coefficient $d_{7}$ dominates the deformation near the fixed end (Z=0), while coefficient $d_{8}$ governs the deformation near the free end (Z=L). These two coefficients serve as the mathematical representation of the end effect in the axial displacement field.

In summary, when investigating the balanced performance of the thick-walled FRF pipe, the semi-analytical method proposed in this study, in comparison with the finite element method that yields discrete numerical solutions adopted in Reference [32], is capable of transforming the complex nonlinear boundary-value problem into a low-dimensional parametric model, while simultaneously accounting for material nonlinearity and the end constraint effect of the finite-length laminated thick-walled pipe. The obtained coefficients of the displacement trial function possess distinct physical significance, which can directly quantify and reveal the dominant deformation mechanisms and thereby deepen the understanding of the deformation behavior of the thick-walled FRF pipe under the self-balanced state. In addition, the semi-analytical method characterizes the mechanical behavior of the pipe as a function of key design variables, including fiber winding angle, material properties, and geometric dimensions. The established parametric analysis can lay a theoretical foundation for the design analysis and optimization of the self-balancing thick-walled FRF pipe.

### 3.4. Effect of the Fiber Winding Angle

The fiber winding angle of the reinforcing fiber is one of the key design parameters that determine the mechanical properties of FRF pipes [33]. For thick-walled FRF pipes, the dependence of axial deformation under the maximum working internal pressure on the fiber winding angle is an issue worthy of in-depth investigation. Analyzing this dependence not only reveals the influence of the fiber winding angle on balanced performance but also verifies the universality of the eight-coefficient displacement trial function across different design parameters.

**Figure 10:**
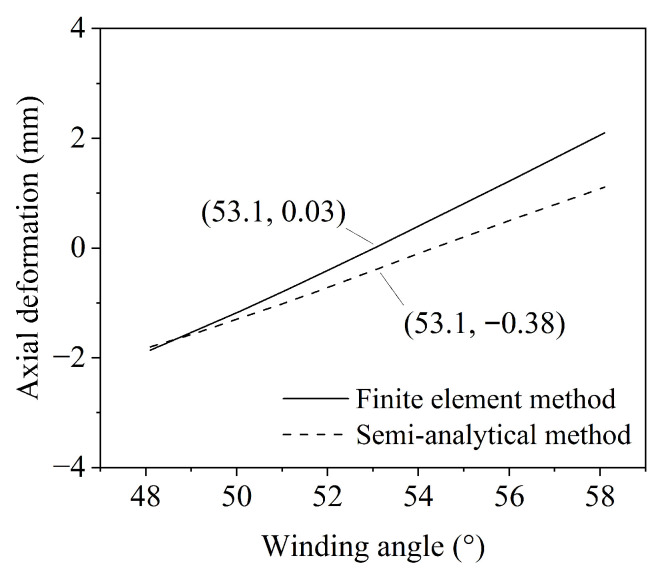
Axial deformation variation of the pipe with changing fiber winding angle.

The axial deformation versus the fiber winding angle curves predicted by the two methods are shown in Figure 10. The results show that the predicted curves from both methods exhibit the same trend. As the fiber winding angle increases, the axial deformation shifts from negative (contraction) to positive (extension), with the minimum deformation occurring near the empirically determined balanced angle.

**Figure 11:**
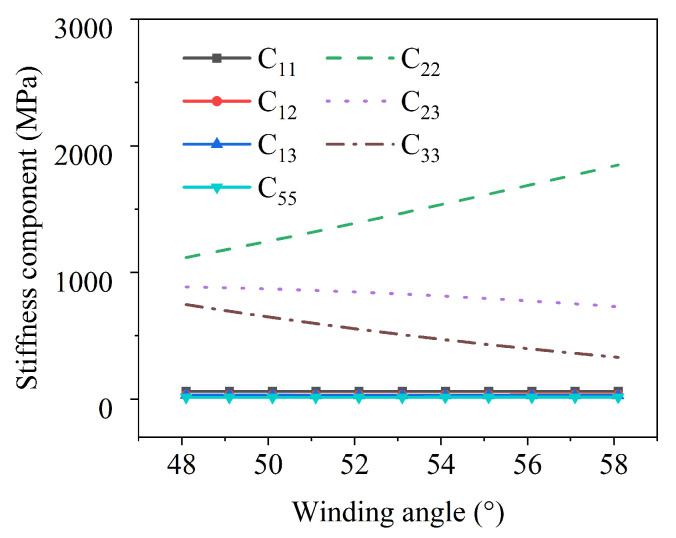
The variation of the reinforcement layer’s stiffness components with changes in fiber winding angle.

According to Formulas (24) and (29) together with the axisymmetric condition, the stiffness components ($C_{14}$, $C_{24}$, $C_{34}$, $C_{44}$, $C_{56}$, and $C_{66}$) in the strain energy density function of the reinforcement layer do not contribute to the deformation. The stiffness components contributing to the axial deformation of the pipe change with the winding angle as shown in Figure 11. Among them, the circumferential stiffness $C_{22}$, the circumferential–axial coupling stiffness $C_{23}$, and the axial stiffness $C_{33}$ change most significantly. As the fiber winding angle increases, $C_{22}$ monotonically increases, while $C_{23}$ and $C_{33}$ monotonically decrease. From a physical perspective, $C_{22}$ can inhibit the circumferential deformation of the pipe, $C_{33}$ constrains the axial deformation of the pipe, and $C_{23}$ regulates the influence of the circumferential strain of the pipe on the axial stress.

The changes in these stiffness components, through interaction with the rubber matrix, dominate the macroscopic axial deformation behavior of the pipe. Based on the characteristic that rubber materials are approximately incompressible, without the reinforcement layer, the radial expansion caused by the internal pressure forces the pipe to undergo axial contraction to meet the requirement of approximately constant rubber volume. Considering the contribution of the reinforcement layer, the fiber winding angle, by changing the equivalent stiffness of the reinforcement layer, modulates the deformation mode of the rubber matrix, thereby determining the macroscopic axial response of the pipe. Near the balanced winding angle, the Poisson effect of the rubber matrix and the reinforcement layer work together, causing the contraction and elongation of the pipe to reach macroscopic balance.

## 4. Conclusions

This paper addresses the engineering requirement of determining the balanced winding angle of FRF pipes prior to manufacturing, aiming to reduce design costs and improve manufacturing efficiency. A semi-analytical method that takes into account end effects, geometric nonlinearity, and material nonlinearity is proposed. The deformation mechanism of the pipe in the balanced state is revealed. The main conclusions are as follows:

1. A novel 8-coefficient displacement trial function capable of characterizing the displacement field of a finite-length thick-walled pipe under internal pressure and end constraints is established. The predicted axial deformation and displacement field from the method show agreement with both experimental results and finite element simulation results;
2. By elucidating the physical meanings of the eight coefficients, the deformation mechanism of the pipe in the balanced state is revealed. Internal pressure induces radial expansion of the pipe matrix, while the reinforcement layer exerts an increasing constraint gradient in the wall thickness direction, and end constraints induce a symmetric warping pattern in axial deformation;
3. Parametric analysis shows that the fiber winding angle of the reinforcing fibers has a decisive influence on the balanced performance of the thick-walled FRF pipe, and that the axial deformation shifts from negative to positive with increasing fiber winding angle. This behavior occurs because the fiber winding angle regulates the axial–radial stiffness distribution in the reinforcement layer while coupling the Poisson effect.

Future research can systematically explore the effects of combinations of different pipe matrices and reinforcing fiber materials, stacking sequences and pipe geometries on the applicability of the method proposed in this paper and on the form of the basis functions. On this basis, it is necessary to promote the transformation of this method from theoretical analysis to an engineering design tool, and verify and improve it through a closed-loop workflow from parameter prediction to performance testing, so as to ultimately enhance its reliability and generality in engineering design.

## Figures and Tables

**Table 1:** The manufacturing parameters of the thick-walled FRF pipe.

Structure Parameter	Value
Length (mm)	166
Inner diameter (mm)	65
Outer diameter (mm)	100
Innermost reinforcement layer diameter (mm)	73
Number of reinforcement layers	4
Reinforcing fiber winding angle (°)	±53.1
Diameter of reinforcing fiber (mm)	0.9
Center-to-center distance of reinforcing fibers (mm)	1.111

**Table 2:** Displacement trial function with varying numbers of undetermined coefficients.

Number of Coefficients	Displacement Trial Function
5	$u_{R}\left(R,Z\right)=\left(d_{1}+d_{2}\frac{R}{R_{in}}\right)\frac{Z}{L}\left(1-\frac{Z}{L}\right)$
	$u_{Z}\left(R,Z\right)=\left(d_{3}\frac{Z}{L}+d_{4}{\left(\frac{Z}{L}\right)}^{2}\right)+d_{5}\frac{R}{R_{in}}\frac{Z}{L}\left(1-\frac{Z}{L}\right)$
6	$u_{R}\left(R,Z\right)=\left(d_{1}+d_{2}\frac{R}{R_{in}}+d_{3}{\left(\frac{R}{R_{in}}\right)}^{2}\right)\frac{Z}{L}\left(1-\frac{Z}{L}\right)$
	$u_{Z}\left(R,Z\right)=\left(d_{4}\frac{Z}{L}+d_{5}{\left(\frac{Z}{L}\right)}^{2}\right)+d_{6}\frac{R}{R_{in}}\frac{Z}{L}\left(1-\frac{Z}{L}\right)$
7	$u_{R}\left(R,Z\right)=\left(d_{1}+d_{2}\frac{R}{R_{in}}+d_{3}{\left(\frac{R}{R_{in}}\right)}^{2}\right)\frac{Z}{L}\left(1-\frac{Z}{L}\right)$
	$u_{Z}\left(R,Z\right)=\left(d_{4}\frac{Z}{L}+d_{5}{\left(\frac{Z}{L}\right)}^{2}\right)+d_{6}\frac{R}{R_{in}}{\left(\frac{Z}{L}\right)}^{2}\left(1-\frac{Z}{L}\right)+d_{7}\frac{R}{R_{in}}\frac{Z}{L}{\left(1-\frac{Z}{L}\right)}^{2}$
8	$u_{R}\left(R,Z\right)=\left(d_{1}+d_{2}\frac{R}{R_{in}}+d_{3}{\left(\frac{R}{R_{in}}\right)}^{2}\right)\frac{Z}{L}\left(1-\frac{Z}{L}\right)$
	$u_{Z}\left(R,Z\right)=\left(d_{4}\frac{Z}{L}+d_{5}{\left(\frac{Z}{L}\right)}^{2}+d_{6}{\left(\frac{Z}{L}\right)}^{3}\right)+d_{7}\frac{R}{R_{in}}{\left(\frac{Z}{L}\right)}^{2}\left(1-\frac{Z}{L}\right)+d_{8}\frac{R}{R_{in}}\frac{Z}{L}{\left(1-\frac{Z}{L}\right)}^{2}$
9	$u_{R}\left(R,Z\right)=\left(d_{1}+d_{2}\frac{R}{R_{in}}+d_{3}{\left(\frac{R}{R_{in}}\right)}^{2}+d_{4}{\left(\frac{R}{R_{in}}\right)}^{3}\right)\frac{Z}{L}\left(1-\frac{Z}{L}\right)$
	$u_{Z}\left(R,Z\right)=\left(d_{5}\frac{Z}{L}+d_{6}{\left(\frac{Z}{L}\right)}^{2}+d_{7}{\left(\frac{Z}{L}\right)}^{3}\right)+d_{8}\frac{R}{R_{in}}{\left(\frac{Z}{L}\right)}^{2}\left(1-\frac{Z}{L}\right)+d_{9}\frac{R}{R_{in}}\frac{Z}{L}{\left(1-\frac{Z}{L}\right)}^{2}$
10	$u_{R}\left(R,Z\right)=\left(d_{1}+d_{2}\frac{R}{R_{in}}+d_{3}{\left(\frac{R}{R_{in}}\right)}^{2}+d_{4}{\left(\frac{R}{R_{in}}\right)}^{3}\right)\frac{Z}{L}\left(1-\frac{Z}{L}\right)$
	$u_{Z}\left(R,Z\right)=\left(d_{5}\frac{Z}{L}+d_{6}{\left(\frac{Z}{L}\right)}^{2}+d_{7}{\left(\frac{Z}{L}\right)}^{3}+d_{8}{\left(\frac{Z}{L}\right)}^{4}\right)+d_{9}\frac{R}{R_{in}}{\left(\frac{Z}{L}\right)}^{2}\left(1-\frac{Z}{L}\right)+d_{10}\frac{R}{R_{in}}\frac{Z}{L}{\left(1-\frac{Z}{L}\right)}^{2}$

**Table 3:** Influence of the number of displacement trial function coefficients on axial deformation.

Number of Coefficients	Axial Deformation (mm)	Adjacent Change Quantity (mm)	The Deviation Relative to the 8-Coefficients (mm)
5	0.12	-	0.5
6	0.09	−0.03	0.47
7	−0.25	−0.34	0.13
8	−0.38	−0.13	-
9	−0.38	0	0
10	−0.38	0	0

**Table 4:** Convergence time for calculations using different displacement trial functions.

Number of Coefficients	Time (s)
8	66.23
9	242.98
10	408.11

**Table 5:** Coefficient values of the 8-coefficient displacement trial function.

Coefficient	Value	Coefficient	Value
$d_{1}$	6.97	$d_{5}$	−10.17
$d_{2}$	−6.54	$d_{6}$	6.78
$d_{3}$	2.11	$d_{7}$	−2.12
$d_{4}$	3.01	$d_{8}$	2.12

## Data Availability

The original contributions presented in the study are included in the article, further inquiries can be directed to the corresponding author.

## Abbreviations

The following abbreviations are used in this manuscript:

FRF	Fiber-reinforced flexible
